# Dexamethasone and levetiracetam reduce hetero-cellular gap-junctional coupling between F98 glioma cells and glial cells in vitro

**DOI:** 10.1007/s11060-016-2324-5

**Published:** 2016-11-16

**Authors:** Fatme Seval Ismail, Zahra Moinfar, Nora Prochnow, Hannes Dambach, Daniel Hinkerohe, Claus Gert Haase, Eckart Förster, Pedro Michael Faustmann

**Affiliations:** 1 Department of Neuroanatomy and Molecular Brain Research, Ruhr University Bochum, Universitätsstr. 150, 44801 Bochum, Germany; 2 International Graduate School of Neuroscience, Ruhr University Bochum, Bochum, Germany; 3 Department of Neurology, University Hospital Knappschaftskrankenhaus Bochum, Ruhr University Bochum, Bochum, Germany; 4 Department of Neurology and Clinical Neurophysiology, Evangelical Hospital Gelsenkirchen, Gelsenkirchen, Germany

**Keywords:** Glioma, Astrocytes, Microglia, Heterocellular gap junctions, Levetiracetam, Dexamethasone

## Abstract

Gap junctions (GJs) in astrocytes and glioma cells are important channels for cell-to-cell communication that contribute to homo- and heterocellular coupling. According to recent studies, heterocellular gap-junctional communication (H-GJC) between glioma cells and their surrounding environment enhances glioma progression. Therefore, we developed a new in vitro model to examine H-GJC between glioma cells, astrocytes and microglia. Consequently, F98 rat glioma cells were double-labeled with GJ-impermeable (CM-DiI) and GJ-permeable dye (calcein AM) and were seeded on unlabeled astrocyte-microglia co-cultures. Dual whole cell voltage clamp recordings were carried out on selected cell pairs to characterize the functional properties of H-GJC in vitro. The expression of four types of connexins (Cxs), including Cx32, Cx36, Cx43 and Cx45, and microglial phenotypes were analyzed by immunocytochemistry. The H-GJC between glioma cells and astrocytes/microglia increased after a longer incubation period with a higher number of glioma cells. We provided evidence for the direct GJ coupling of microglia and glioma cells under native in vitro conditions. In addition, we exploited this model to evaluate H-GJC after incubation with levetiracetam (LEV) and/or dexamethasone (DEX). Previous in vitro studies suggest that LEV and DEX are frequently used to control seizure and edema in glioma. Our findings showed that LEV and/or DEX decrease the number of heterocellular coupled cells significantly. In conclusion, our newly developed model demonstrated H-GJC between glioma cells and both astrocytes and microglia. The reduced H-GJC by LEV and DEX suggests a potential effect of both drugs on glioma progression.

## Introduction

Gap junctions (GJs) in astrocytes and glioma cells are important channels that contribute to cell-to-cell communication allowing the exchange of small molecules, ions and second messengers, and ensuring the homeostasis in multicellular systems [1]. Connexin 43 (Cx43) is the predominant GJ protein in astrocytes and in glioma cells [1]. In addition to gap junctional communication (GJC) within a homogenous cell population, astrocytes and glioma cells are able to interact with each other through heterocellular GJ communication (H-GJC) [2, 3] that might support glioma invasion, adhesion and migration [3–5].

Microglia cells are immune cells of the central nervous system. Ramified microglia represent the predominant morphology in the healthy brain, ranging from 5 to 20% of the glial cell population [6]. After activation by foreign pathogens [7], e.g. glioma cells, microglia proliferate and undergo morphological changes towards a rounded phagocytic type, express immune molecules and release inflammatory mediators [8]. Studies show that microglial cells can express Cx43 and communicate with each other through GJs under inflammatory conditions [9].

Brain edema and seizures are the most common complications of glioma. Levetiracetam (LEV) and dexamethasone (DEX) are commonly administered to control these symptoms. Levetiracetam belongs to the second generation broad-spectrum anticonvulsants and, due to fewer side effects in comparison to other anticonvulsants, is preferred as a first-choice drug in glioma-associated seizure [10]. Moreover, LEV shows anti-inflammatory properties in astrocytic cultures [11, 12]. Dexamethasone is a highly potent anti-inflammatory and anti-edematous steroid with regulatory effects on microglia morphology [13] and glioma cell proliferation [14]. The effects of both drugs on homocellular GJC have been separately investigated in glioma cells and in primary astrocytes in vitro [11–13]. However, the effects of LEV and DEX on H-GJC have not yet been studied. Therefore, we developed an in vitro co-culture model of astrocyte-microglia-glioma cells to study the effects of LEV and DEX on H-GJC. Subsequently, we evaluated: (1) the H-GJC between these cells with regard to the number and the incubation period of tumor cells in microglia-astrocyte cultures; (2) the physiological properties of heterocellular trans-junctional coupling between glioma cells and microglia by the use of dual whole cell voltage clamp recordings; (3) Cx32, Cx36, Cx43, Cx45 expressions on glioma cells; and (4) modulations of microglial morphology by immunocytochemistry.

## Materials and methods

### Cell culture

Primary astrocyte-microglia cultures were prepared from cerebral hemispheres of postnatal (P0–P2) Wistar rats [6]. Briefly, cerebellum, meninges and choroid plexus were removed and the brains were kept in ice-cold phosphate-buffered saline (PBS). Following treatment with 0.1% trypsin (Invitrogen) for 30 min at 37 °C, centrifugation at 200×*g* for 5 min was performed to remove the supernatant. After incubation with 1% DNAse I (Serva) for 5 min at room temperature (RT), adding cell culture medium (Dulbecco’s minimal essential medium, DMEM, with 1% glucose, 10% fetal calf serum, 1% nonessential amino acids (Invitrogen), penicillin (50 µg/ml), streptomycin (50 µg/ml) and glutamine (2 mM)) and centrifugation, they were filtered through a 60-µm nylon mesh and inserted in a plastic tissue-culture flask (Falcon). The flasks were kept in 7% CO_2_, 93% air atmosphere at 37 °C at almost 100% relative humidity. This method achieved astrocyte-microglia cultures depleted of other cell types including a negligible amount of oligodendrocytes and almost 0% of neurons (supplement data from [15]). After 4–5 days, the cultures reached about 100% confluent and were placed on poly-L-lysine-coated glass cover slips (113 mm^2^) at 70,000 cells per well in 24-well plates. F98 rat glioma cells (ATCC^®^) were kept in minimum essential medium (MEM) and placed with two different densities of cells (4000 or 40,000) on the confluent background of astrocyte-microglia.

### Treatment of cultures

Levetiracetam (Sigma, 50 and 100 µg/ml), DEX (Sigma, 1 and 10 µM) or a combination of both (LEV 50 µg/ml + DEX 1 µM or LEV 100 µg/ml + DEX 10 µM) were applied to the astrocyte-microglial-F98 co-cultures for 24 h. Concentrations of DEX and LEV were chosen based on previous studies and minimum effective concentration of each drug [13, 16, 17]. A minimum of six independent experiments were studied for each concentration.

### Assessment of functional H-GJC

In order to test whether glioma cells, astrocytes and microglia communicate via heterocellular GJs, we used a preloading technique [18, 19] by double labeling F98 cells before seeding on astrocyte-microglia cultures in 24-well plates. For this purpose, 1 × 10^6^ F98 cells/ml were simultaneously incubated with the GJ-permeable dye, calcein AM (5 µM; Molecular Probes) and the impermeable cell membrane marker Chloromethyl-benzamidodialkylcarbocyanine (Vybrant CM-DiI cell-labeling solution; 5 µl/ml; Molecular Probes) at 37 °C for 40 min (under these conditions 100% labeling of glioma cells) [18, 19] and placed on unlabeled astrocyte-microglia cultures at the ratio of 1:45 or 1:4.5 (4000 or 40,000, respectively). The cultures were evaluated after 5 or 24 h incubation. The cover slips were fixed with 4% paraformaldehyde (PFA) and counterstained with ProLong^®^ Gold Antifade Reagent (Molecular Probes) to assess H-GJC. We could differentiate labeled tumor cells from astrocyte and microglia due to different excitation wavelength of calcein and CM-DiI. Moreover, we were able to track the dye transfer from tumor cells to the surrounding microglia by labeling microglia with polyclonal ionized calcium-binding adapter molecule 1 antibody (Iba1, 1:300, Wako Pure Chemical Industries). The heterocellular coupling index (HCI) was characterized by calculating the mean of the number of adjacent calcein-labeled astrocyte-microglia in the direct surroundings of one double-labeled glioma cell. The H-GJC was analyzed by fluorescence (Zeiss, Axiovert 35 microscope) and confocal scanning microscopy (Zeiss LSM 510, inverted confocal microscope) at 630× magnification in a minimum of four independent experiments.

### Immunocytochemistry

Cover slips with F98 monocultures and mixed astrocyte-microglia-F98 cell cultures were fixed with 4% PFA and incubated in PBS-blocking solution containing 10% horse serum and 1% bovine serum albumin. The cover slips were treated with poly-/monoclonal rabbit anti-Cx32, anti-Cx36, anti-Cx43, anti-Cx45 (1:200, 1:50, 1:2000, 1:50; Zymed, Sigma, Chemicon) and polyclonal rabbit anti-Iba1 (1:300) and incubated at 4 °C overnight. The next day, the wells were incubated with secondary antibodies (1:500, Molecular Probes) including goat anti-rabbit IgG conjugates (Alexa fluor^®^ 633) and goat anti-rabbit IgG conjugates (Alexa fluor^®^ 488) for 45 min. Immunocytochemically labeled cells were counterstained with DAPI (1:2500) to quantify the cell numbers. The microglia morphology was evaluated in a minimum of six different visual fields on each cover slip. Iba1 staining allowed the classification of microglia as ramified, intermediate and activated rounded phagocytic phenotype [6]. The percentage of each subpopulation was calculated based on the ratio to the whole population of microglia.

### Electrophysiology

#### Dual whole cell patch clamp recording

The dual whole cell voltage clamp recordings were used to identify macroscopic currents from potentially electrically coupled cultured amoeboid microglia and F98 glioma cells. Current recordings took place in a recording chamber (volume: 1 ml) mounted on an inverted microscope (Zeiss Axiovert; Carl Zeiss AG). Cells were visualized by phase contrast microscopy and continuously superfused (4 ml/min) with oxygenated standard cerebrospinal fluid (according to [20, 21]), containing (in mM): 124 NaCl, 2.69 KCl, 1.25 KH_2_PO_4_, 26 NaHCO_3_, 2 MgSO_4_, 2 CaCl_2_ and 10 glucose (Sigma) at RT. Borosilicate glass patch pipettes were pulled to an input resistance of 2–5 MΩ. Electrodes of less than 5 MΩ and cell pairs with initial conductance less than 10 nS were used exclusively in the calculations of the gating properties. Trans-junctional currents (Ij) were measured in one cell of the pair (clamped to constant voltage), while voltage steps (from −120 to 20 mV; 10 mV increments) of 25 s duration were applied to the neighboring cell. All signals were recorded in the voltage clamp mode, amplified and digitized at 11 kHz and acquired at 1 kHz for analysis by two Axopatch 200B amplifiers (Axon Instruments; Molecular Devices). Series resistance and capacitance were compensated (40–50%) for improved voltage clamp control. Data were digitized, displayed and analyzed using WinWCP (Strathclyde; Biologic). A 16-bit analogue to digital converter (BNC 2110 connected to Ni­PCi 6229, National Instruments) was used to digitize the signals.

### Data analyses and statistics

The differences between the means of the groups were analyzed for H-GJC assessment and microglia morphological study by the one-way ANOVA (F, p), using the Bonferroni post hoc test for parametric data or Kruskal-Wallis and Dunn’s test for nonparametric data. All statistical analyses and graphs were performed with GraphPad Prism version 5.00 for Windows (GraphPad Software) or OriginPro (Origin Corporation). Sigma Plot 11.0 (Systat Software, Chicago, USA) was used to analyze the induced effects on membrane current response properties dependent on the voltage protocol condition. Comparisons between two data sets were carried out using an unpaired nonparametric Mann-Whitney rank sum comparison. The significance was set at p < 0.05 and the results were reported as mean ± standard error of the mean.

## Results

### H-GJC between malignant glioma cells and astrocytes/microglia

**Fig. 1:**
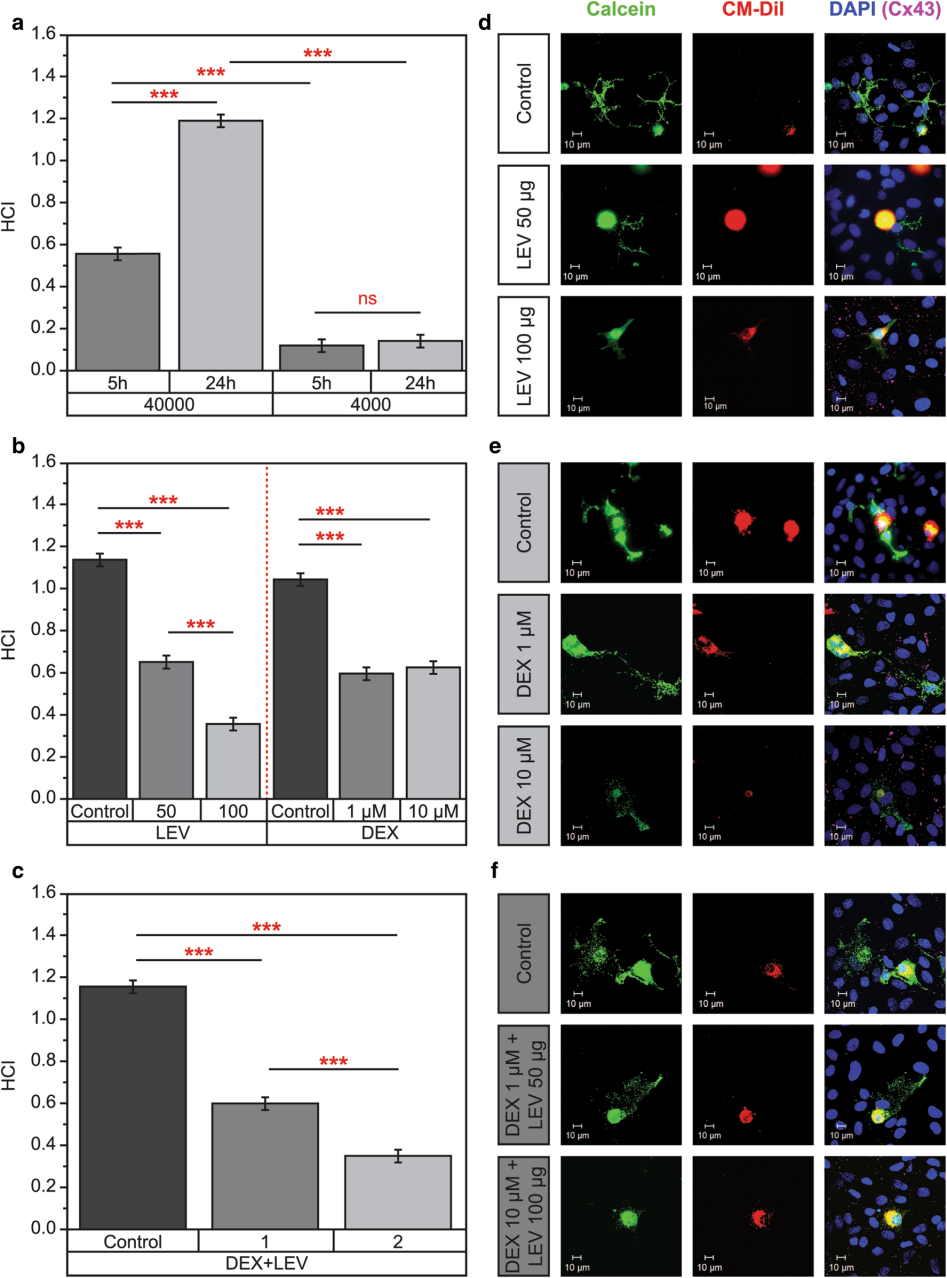
Heterocellular coupling of F98 glioma cell line with astrocytes/microglia. **a** Significant difference of H-GJC in cultures with high (40,000) and low (4000) F98 cells after 5 or 24 h incubation. **b, c** H-GJC between F98 glioma cells and astrocytes/microglia was decreased significantly under LEV and/or DEX. Data were collected by at least four independent experiments and tested with Kruskal-Wallis and Dunn’s post hoc test, *p < 0.05, **p < 0.01, ***p < 0.001. *LEV* levetiracetam, *DEX* dexamethasone, *HCI* heterocellular coupling index (number of recipient astrocytes/microglia per one F98 tumor cell), *1* DEX 1 μM + LEV 50 µg/ml, *2* DEX 10 μM + LEV 100 µg/ml. Units for 50 and 100 LEV in **b** are not indicated (µg/ml). **d**–**f** Reduced heterocellular coupling after incubation of the astrocyte-microglia-F98 cultures with DEX and/or LEV is demonstrated by immunocytochemistry. Functional H-GJC is visualized by transfer of calcein from CM-DiI-labeled F98 donor cells to unlabeled recipient astrocytes/microglia. Cell nuclei were detected by applying DAPI. Calcein: *green*, CM-DiI: *red*, Cx43 (not in all pictures visible): *pink*, DAPI: *blue*, merged picture

The GJ coupling between F98 glioma cells and astrocyte/microglia was very low in cultures with a low number of F98 glioma cells (4000) after 5 or 24 h of incubation (HCI: 0.1 ± 0.02, n = 4, p = ns). The HCI increased significantly in the cultures with higher numbers of F98 cells (40,000) at 5 h (0.6 ± 0.03, p < 0.001) and 24 h (1.2 ± 0.02, p < 0.001) compared to 4000 cells (Fig. 1a). The low number of F98 cells in the co-cultures showed a very low HCI. Similarly, shorter incubation with either a high or low number of F98 cells did not cause significant coupling between glioma cells and their surroundings. Therefore, we utilized mixed cultures of 40,000 F98 for the experiments with drug incubations and reported results after 24 h incubation.

Since functional GJC through heterotypic Cx proteins on adjacent cells is not likely [22] and different types of Cxs are expressed on either astrocytes or microglia, we evaluated the expression of Cx43, Cx32, Cx36 and Cx45 in glioma cells to ensure the type of probable GJC between these cells. Our results showed that Cx43 was the single GJ that was predominantly expressed on F98 glioma cells.

### LEV and/or DEX significantly decreased the H-GJC between F98 glioma cells and astrocytes-microglia

The HCI was reduced from 1.1 ± 0.02 in the control group to 0.6 ± 0.03 under 1 μM and to 0.6 ± 0.03 under 10 μM DEX (p < 0.0001, n = 6) (Fig. 1b, e). Similarly, the HCI was decreased (p < 0.0001, n = 6) significantly from 1.1 ± 0.02 in the untreated control group to 0.6 ± 0.04 under 50 µg/ml LEV and to 0.4 ± 0.04 under 100 µg/ml LEV (p < 0.0001, n = 6). The effect of LEV on H-GJC was dose-dependent (Fig. 1b, d).

The HCI was decreased (p < 0.0001, n = 6) significantly in cultures incubated with 1 μM DEX in combination with 50 µg/ml LEV (0.6 ± 0.03) compared to the untreated controls (1.2 ± 0.02). Similarly, the combination of 10 μM DEX and 100 µg/ml LEV reduced the HCI significantly to 0.4 ± 0.03 compared to the control (1.2 ± 0.02, p < 0.0001, n = 6). The effect of the combination of both drugs on the HCI was dose-dependent (Fig. 1c, f).

### Voltage dependence of macroscopic junctional currents between F98 glioma cells and microglia

**Fig. 2:**
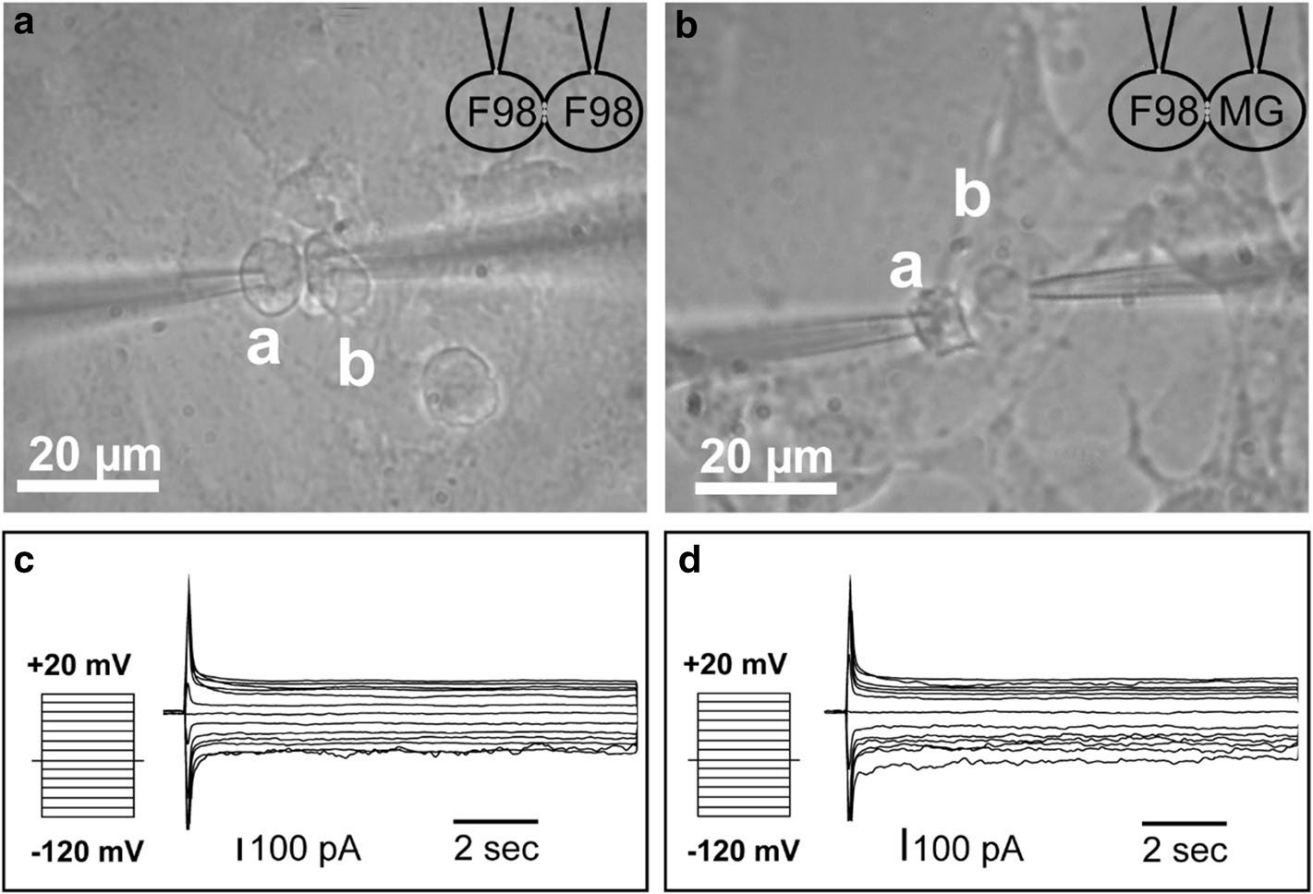
Homo- and heterocellular gap junction (H-GJC)-based coupling properties characterized in dual whole cell patch clamp recordings in the voltage clamp mode. **a** Sample pair of F98 glioma cells (F98). **b** Amoeboid microglia (MG) coupled to an F98 glioma (F98) cell during recording. **c, d** Corresponding trans-junctional current responses of the sample pairs in **a** and **b** following stepwise holding potential depolarization from −120 to +20 mV condition (each 10 mV increment; 25 s pulse duration) while the neighbor (*b*) was kept at −60 mV

In order to evaluate the physiological properties of homo- and heterocellular trans-junctional coupling, dual whole cell voltage clamp recordings were performed on pairs of F98 cells (n = 5) (sample pair Fig. 2a) and amoeboid microglial cells linked to F98 glioma cells (n = 5) (sample pair Fig. 2b). One cell (a in Fig. 2) was patched and directed to stepwise holding potential depolarization from either −120 to +20 mV (10 mV increment; 25 s pulse duration), while the neighbor (b in Fig. 2) was kept at −60 mV for subsequent trans-junctional current recordings in the whole cell configuration for data acquisition. Junctional currents (Fig. 2c) were inactivated when trans-junctional voltages from −120 to +20 mV were applied for the homocellular constellation of coupled F98 cells (Fig. 2a). While the initial conductance remained unchanged by voltage, the steady state conductance decreased for trans-junctional voltage values larger than −40 mV. Similar findings were made for heterocellular pairs of F98 glioma and microglial cells (Fig. 2b, d). The electrophysiological data provides clear indication that active shaped microglia are capable of forming electrical synapses with F98 cells in vitro. This finding is in alignment with the observations from our immunocytochemical studies. Because of the significant reduced and very low hetero-cellular coupling after incubation with DEX and/or LEV demonstrated by immunocytochemistry, as shown in Fig. 1b–f, there are obviously no visible couplings and as a result no available data for dual whole cell voltage clamp recordings under DEX/LEV conditions.

### Microglial phenotypes in astrocyte-microglia-glioma cell co-cultures

**Fig. 3:**
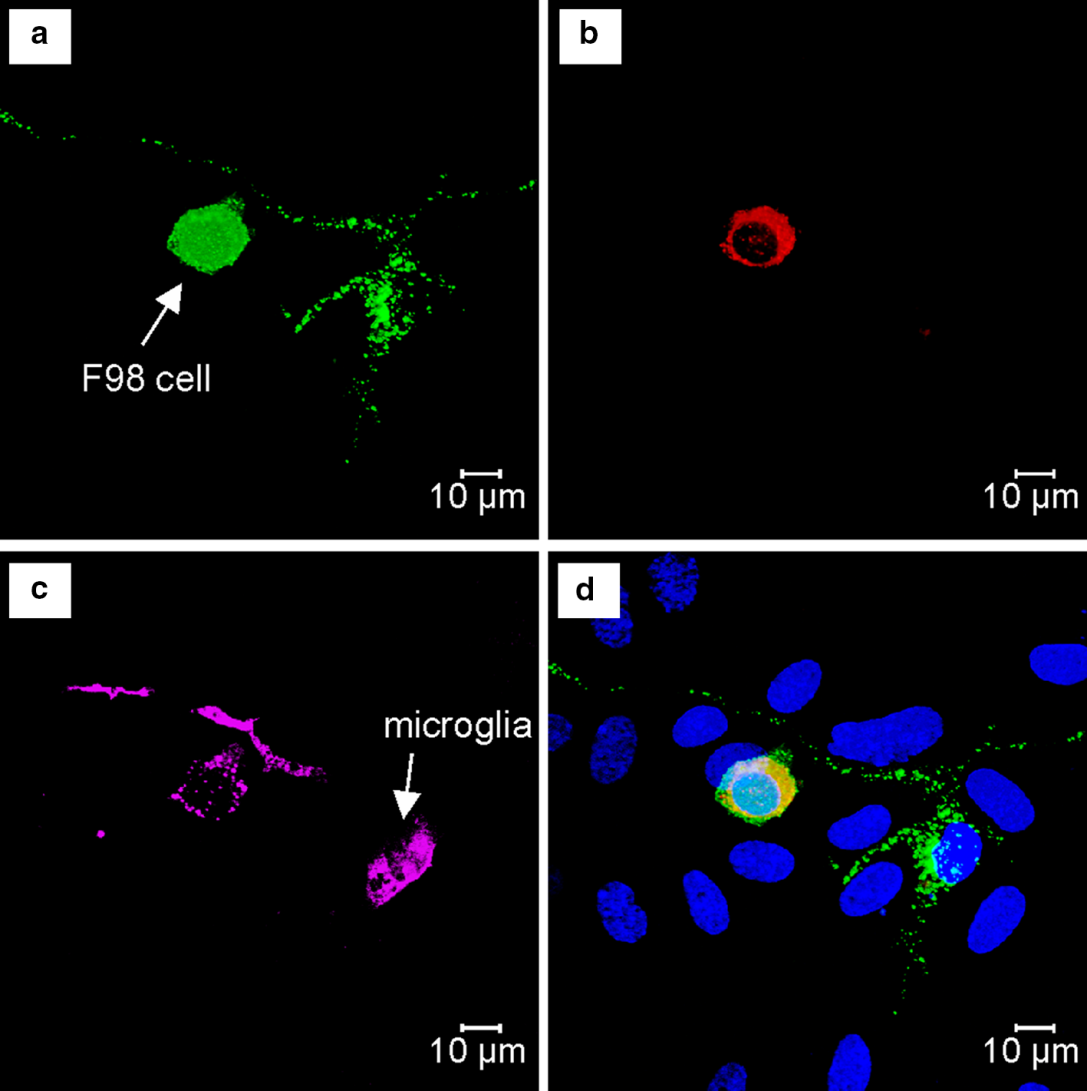
H-GJC between F98 cells and microglia in the cultures of astrocyte-microlgia-F98 cells. Microglial cells were marked with Iba1. Functional H-GJC is visualized by transfer of calcein from CM-DiI-labeled donor cells to unlabeled recipient astrocytes and microglia. Cell nuclei were detected by applying DAPI. Note that F98 cells express Iba1. **a** Calcein *green*. **b** CM-DiI *red*. **c** Iba1 *pink*. **d** DAPI *blue*, merged picture

The addition of different numbers of F98 tumor cells (4000 or 40,000 cells) to the astrocyte-microglia co-cultures for 5 and 24 h did not alter the microglial morphology compared to the glioma cell-free control groups (data not shown). The entire microglial cell number in both control groups (5 h: 5 ± 1%; 24 h: 9 ± 1%) was not changed significantly after incubation with either 4000 or 40,000 glioma cells for 5 or 24 h (p = ns; data not shown). Although F98 cells were negative for Iba-1 in monocultures, F98-glioma cells, which were incubated in the presence of astrocytes and microglial cells, expressed Iba-1 (Fig. 3).

## Discussion

In this study, we developed a new in vitro co-culture model to investigate the H-GJC between glioma cells and astrocytes/microglia and to evaluate the effects of DEX and LEV on heterocellular coupling.

### Functional hetero-cellular gap-junctional coupling between glioma cells and astrocytes/microglia

Since GJs are implicated in cell growth and differentiation, modulations of GJs on glioma cells have been investigated in several experiments [23, 24]. Previous studies demonstrated the heterocellular coupling between astrocytes and glioma cells [2, 3]; however, H-GJC has not been studied in the presence of microglia in vitro. Our in vitro study showed that the number of F98 cells is an essential factor for H-GJC. A reasonable answer to this effect might be the accumulating effect of soluble factors, such as cytokines from F98 cells, which is obviously enhanced with increasing cell numbers in the culture.

Our results showed that Cx43 is the predominant Cx in F98 cells. On the other hand, despite the controversial studies about the expression of Cx43 on microglia [9, 25], the expression of Cx32 and Cx36 on microglia has been shown previously [26]. Using dual whole cell patch clamp recording and immunocytochemistry, we demonstrated that microglia are capable of forming electrical synapses with F98 cells in vitro. We believe that Cx43 is the major GJ in H-GJC between microglia and F98 cells in our study because: (1) Cx43 is the predominant GJs on F98 cells, whereas the expression of Cx32, Cx36 and Cx45 in F98 glioma cells were negative (data not shown), (2) only homotypical GJCs are functional [22], and (3) the trans-junctional current amplitudes, conductance and activation pattern give rise to Cx43-based properties.

Some F98 tumor cells showed significantly increased Iba-1 immunoreactivity after incubation with astrocyte-microglia co-cultures compared to the monoculture of tumor cells. Since GJs allow the rapid transfer of gene fragments through a cellular network [27, 28], such DNA-transfer between microglia and glioma cells may contribute to Iba-1 expression in glioma cells. This transfer could be one of the immune escape mechanisms that leads to incapacity of the immune system to detect and reject transformed cells. The H-GJC between glioma cells and microglia might represent one of the underlying mechanisms contributing to glioma cell migration [29]. These observations could be useful in the development of novel immunotherapy strategies.

### DEX and LEV decreased H-GJC between F98 cells and astrocytes/microglia

Dexamethasone is commonly used as symptomatic therapy for tumor-associated edema [30] and LEV is applied as one of the first therapeutic choices for seizures in glioma. However, little is known about their probable effects on glioma cells. Previous studies have shown that DEX inhibits proliferation of glioma cells [14] and reduces both their migration and invasion [31]. Although the mechanism of such effects is not well understood, probable mechanisms, such as reduced matrix metalloproteinase-2 secretion from glioma cells [31], decreased Cx43 expression in glioma cells [13] or reduced microglia recruitment and activation [32], have been proposed. Based on our results, decreased H-GJC between F98 cells and astrocytes/microglia might be a new mechanism of effect of DEX in glioma progression.

In addition to anticonvulsant activity, LEV can contribute a positive effect to the specific glioma treatment [10] and exhibits anti-inflammatory properties in vitro [11]. Taking into account that GJC has been implicated in seizure pathogenesis, disconnection of H-GJC caused by LEV and/or DEX could have a possible role in reducing seizure activity.

We could not detect specifically whether HCI reduction by LEV and DEX originated from the H-GJC of astrocyte-F98 or microglia-F98 cells. However, since astrocytes outnumber microglia in our co-cultures, we speculated that the reduction of H-GJC by LEV and DEX reflects astrocyte-F98 GJC. Our findings generally suggest an additional mechanism besides anti-inflammatory and anticonvulsant activity through which DEX and LEV might affect seizures and glioma progression as well.

## Conclusion

We developed a novel in vitro co-culture model of astrocytes, microglia, and glioma cells to study the effects of DEX and LEV on H-GJC between glioma cells and astrocytes-microglia co-cultures. We demonstrated the direct functional H-GJC between glioma cells and microglia. In our study, DEX and LEV decreased H-GJC between glioma cells and astrocytes/microglia. This new aspect could point to a potential effect of DEX and LEV on glioma migration and seizure activity. Additional studies using clonal variation in tumor genicity and cell growth, and in vivo animal models are required to evaluate the impact of current findings under DEX and LEV treatment. Our model allows the testing of aspects of glioma in response to frequently applied drugs. Additionally, it can be used in the relevant research area to identify the cell biological processes imposed by glioma cells.
